# Utilization of iPad in the system of emergency demand and acceptance

**DOI:** 10.1186/cc11087

**Published:** 2012-03-20

**Authors:** K Yamada, Y Sakamoto, Y Enjiyouji

**Affiliations:** 1 Saga University, Saga, Japan; 2 Saga Prefectural Government, Saga, Japan

## Introduction

This study reports that the transportation time by ambulance was shorter following the introduction of iPad (Apple, Inc.) to the current system of emergency demand and acceptance in Saga Prefecture, Japan. There were about over 5,000,000 ambulance dispatches in Japan, and the time for transportation is increasing (the national average: 36.1 minutes) [1]. The administration has made various efforts nationwide that did not achieve any positive results. Although the information system of medical institutions and the emergency medical service (99 Saga Net) was established in 2003 in Saga, it has been underutilized. The Saga prefectural government renewed the previous system as the real-time system of emergency demand and acceptance for the first time in Japan in April 2011.

## Methods

Cloud computing has provided new system to facilitate Internet access from ambulances. In addition, iPads were put into all ambulances (about 55) and emergency medical technicians can get the picture of acceptable hospitals in real time. Emergency personnel who arrive on the scene select the patient's symptoms with an iPad, and this new system displays an up-to-date list of acceptable hospitals. The data that the emergency personnel entered into the system from the iPad are uploaded to 99 Saga Net immediately. Therefore, both the emergency personnel and medical staff in the hospital share the information of where the emergency occurred, the transportation and the medical institutes to which patients were transferred in real time.

## Results

The transportation time by ambulance was shorter for the first time since statistics were first kept in 1999, the mean time was 33.7 minutes in 2009 and 33.2 minutes in April 2011. Furthermore, the new system is expected to reduce the operational costs by 40,000,000 yen a year. The data on the transportation time by ambulance are continually stored in the system and analyses are continuing.

## Conclusion

The introduction of iPad to the new 99 Saga Net has three beneficial points. First, the utilization of information and communication technology is useful for a realistic emergency medical setting. Second, the situation of a realistic emergency medical setting is visualized in real time. Finally, both the emergency personnel and the medical staff in the hospital share the information in an emergency medical setting by eliminating vertically divided administrative functions. Medical personnel will work with local governments in the future to analyze the data from this new system.
